# Towards the Enhancement of Essential Oil Components’ Antimicrobial Activity Using New Zein Protein-Gated Mesoporous Silica Microdevices

**DOI:** 10.3390/ijms22073795

**Published:** 2021-04-06

**Authors:** Elisa Poyatos-Racionero, Gemma Guarí-Borràs, María Ruiz-Rico, Ángela Morellá-Aucejo, Elena Aznar, José Manuel Barat, Ramón Martínez-Máñez, María Dolores Marcos, Andrea Bernardos

**Affiliations:** 1 Instituto Interuniversitario de Investigación de Reconocimiento Molecular y Desarrollo Tecnológico (IDM), Universitat Politècnica de València and Universitat de València, Camino de Vera s/n, 46022 Valencia, Spain; elpora@upvnet.upv.es (E.P.-R.); gem_gnde@hotmail.com (G.G.-B.); ngemoau@upvnet.upv.es (Á.M.-A.); elazgi@upvnet.upv.es (E.A.); rmaez@qim.upv.es (R.M.-M.); 2 CIBER de Bioingeniería, Biomateriales y Nanomedicina (CIBER-BBN), 46022 Valencia, Spain; 3 Departamento de Tecnología de Alimentos, Universitat Politècnica de València, Camino de Vera s/n, 46022 Valencia, Spain; maruiri@etsia.upv.es (M.R.-R.); jmbarat@tal.upv.es (J.M.B.); 4 Unidad Mixta de Investigación en Nanomedicina y Sensores, Universitat Politècnica de València, Instituto de Investigación Sanitaria La Fe (IISLAFE), Av Fernando Abril Martorell 106, 46026 Valencia, Spain; 5 Unidad Mixta UPV-CIPF de Investigación en Mecanismos Enfermedades y Nanomedicina, Universitat Politècnica de València, Centro de Investigación Príncipe Felipe, C/ Eduardo Primo Yúfera 3, 46012 Valencia, Spain; 6 Departamento de Química, Universitat Politècnica de València, Camino de Vera s/n, 46022 Valencia, Spain

**Keywords:** mesoporous silica particles, zein, corn, molecular gate, protease secretion, *E. coli*, foodborne illnesses, food preservative, essential oil components, cinnamaldehyde

## Abstract

The development of new food preservatives is essential to prevent foodborne outbreaks or food spoilage due to microbial growth, enzymatic activity or oxidation. Furthermore, new compounds that substitute the commonly used synthetic food preservatives are needed to stifle the rising problem of microbial resistance. In this scenario, we report herein, as far as we know, for the first time the use of the zein protein as a gating moiety and its application for the controlled release of essential oil components (EOCs). The design of microdevices consist of mesoporous silica particles loaded with essential oils components (thymol, carvacrol and cinnamaldehyde) and functionalized with the zein (prolamin) protein found in corn as a molecular gate. The zein protein grafted on the synthesized microdevices is degraded by the proteolytic action of bacterial enzymatic secretions with the consequent release of the loaded essential oil components efficiently inhibiting bacterial growth. The results allow us to conclude that the new microdevice presented here loaded with the essential oil component cinnamaldehyde improved the antimicrobial properties of the free compound by decreasing volatility and increasing local concentration.

## 1. Introduction

Foodborne outbreaks caused by ingestion of food contaminated with pathogenic microorganisms are among the main threats to public health and the biggest impediments to socioeconomic development all over the world. Food poisoning caused by pathogens or their toxins have been established as the main source of foodborne illnesses in both developing and developed nations. Although some viruses, prions or protozoa can be the cause of these illnesses, most of them are caused by fungi and bacteria. Some examples of foodborne germs are specific strains of *Salmonella enterica*, *Listeria monocytogenes*, *Clostridium botulinum or Escherichia coli*, among others [1].

Pesticides, food preservatives and antibiotics have been generally used with the aim of fighting the pathogenic microorganisms that cause foodborne outbreaks. Occasionally, they have been used indiscriminately, generating an even greater problem, i.e., antimicrobial resistance [2,3]. In this context, the development of new alternatives is necessary to solve this increasing problem of public health. With this aim, numerous studies have focused on the potential use of natural compounds due to the problems derived from common preservatives and their rejection by consumers [4,5,6].

As a suitable option, general attention has been turned to the study of the versatile properties of essential oil components (EOCs) as a possible alternative to synthetic antimicrobial agents [7,8]. EOCs are a wide group of bioactive chemical components found naturally in essential oils [9]. These essential oils are complex mixtures of 20–60 molecules produced during secondary metabolism of aromatic and medicinal plants with important antioxidant, bactericidal, fungicidal or antiparasitic effects [10]. Due to their numerous properties, EOCs have been used since the beginning of humankind and are still being used nowadays as an alternative to other synthetic chemical compounds in the pharmaceutical and food industry [11,12].

The antimicrobial action of EOCs can be attributed to a set of cascade reactions that results in the destruction of a bacterial cell [13]. Several mechanisms have been described to explain the antimicrobial action of EOCs, one of the most relevant being the destabilization of lipids of the cell membrane and the mitochondria due to the hydrophobic character of EOCs [14]. However, other mechanisms can also occur that finally result in the microorganism’s death, such as damage to membrane proteins or inhibition of the electron transport chain, among others [13].

However, EOCs’ high volatility, high reactivity and low solubility in water limit their application [15,16]. Several strategies have been investigated to improve the use of EOCs, most of them aiming to reduce their volatility and increase their solubility. Among these strategies, the use of encapsulation protocols has been explored [14,17,18]. Among the materials for EOC encapsulation, mesoporous silica particles (MSPs) present several advantages, such as high pore volume for loading molecules, easy chemical functionalization and low toxicity levels [19]. In fact, MSPs have been used for the protection and controlled release of (i) bioactive molecules for the improvement of organoleptic or nutritional properties of foods [20] or of (ii) antimicrobial agents to design novel preservatives [18,21,22]. Moreover, given the easy chemical modification of the MSPs’ surface, a wide variety of organic moieties have been used as molecular gates (also known as gatekeepers or nanovalves) to develop advanced delivery systems [23,24,25]. In these systems, the gated materials can ideally show a “zero” payload release, yet the presence of predefined physical, chemical or biochemical stimuli induce pore opening and cargo delivery. This concept has been widely applied to drug delivery [26,27], sensing [28,29] and communication protocols [30,31,32]. Among gating moieties, proteins have been employed as pore-blocking agents in hybrid MSP materials, allowing entrapped cargo release when proteolytic enzymes are present [33,34,35,36,37,38,39,40]. However, considering the inherent presence of bacterial protease secretion as a potential stimulus for cargo release, studies in the literature aiming to develop protein-capped antimicrobial carriers are still scarce [41,42]. Moreover, protease secretion is a key factor in the virulence of pathogen growth [43], so the greater the pathogen virulence, the larger the protease secretion and therefore the higher the triggering stimulus. Based on the aforementioned premises, the attainment of a protein able to act as a capping moiety is preeminent to develop alternative MSP-based antimicrobial systems. In this scenario, a low-cost, industrially produced and widely studied protein is α-zein, the most abundant protein in corn [44,45,46,47]. Alpha-zein (hereinafter referred to as “zein”) is an approximately 22-kDa prolamin protein with a complex tertiary structure which strongly depends on the solvent, based on nine repeating α-helices, with an elongated proposed shape [47]. Although zein is a widely described protein and it has even been studied as a matrix for the preparation of antibacterial nanocomposites including essential oil-loaded MSPs [48], its use specifically as a gatekeeper for MSPs has not been reported yet. However, its own characteristics make zein a viable option to block pores in MSPs and, therefore, to prepare enzyme-responsive particles to protect and actively release EOCs. Furthermore, as the zein protein is a food grade compound, it can be applied for fortification in food systems. Thus, EOCs’ volatility can be reduced through their encapsulation into MSPs, and their active controlled release triggered by proteolytic enzymes can enhance their antimicrobial action, improving EOCs’ bioavailability, with the aim of increasing food safety while extending the shelf life of food.

Based on the above, we report herein for the first time, as far as we know, the application of the zein protein as a gating moiety and its application for the controlled release of EOCs (thymol, carvacrol and cinnamaldehyde). Additionally, we evaluate here the antimicrobial activity of the prepared zein-gated MSPs against *E. coli* as common foodborne bacteria.

## 2. Results and Discussion

### 2.1. Design, Synthesis and Characterization of Gated Systems

**Scheme 1:**
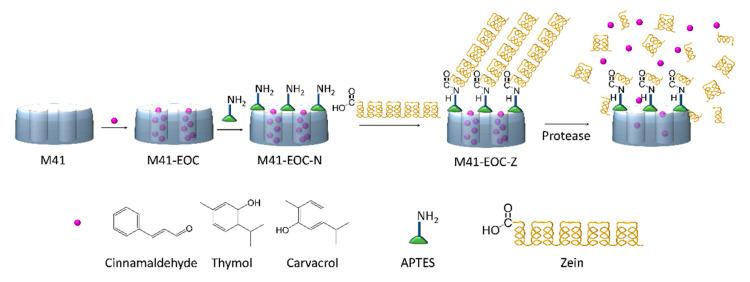
Representation of M41–EOC–Z synthesis and cargo release. Cargo loading (EOCs) is followed by APTES-functionalization, and finally zein is attached to the amino groups. The cargo is actively released due to the hydrolyzing action of protease enzymes.

The design of smart delivery systems is based on three components: (i) a porous support, (ii) bioactive compounds with antimicrobial properties and (iii) a biomolecule as a molecular gate or gatekeeper. MCM-41 mesoporous silica material was selected as inorganic support due to its loading capacity and easy-to-functionalize surface [19]. Essential oil components (EOCs) were chosen as cargo because they are naturally occurring antimicrobial compounds considered safe in the food industry [49]. In addition, zein (corn protein) [47] was selected as the gatekeeper. Following this approach, three materials were prepared (M41–EOC–Z solids), all of them using mesoporous silica microparticles loaded with three different EOCs (i.e., Thy, Car and Cin) and capped with the zein protein (Scheme 1). The preparation of the materials was carried out in three consecutive stages. The first step consisted in loading the mesoporous material with the EOC by vapor adsorption. Next, the functionalization of the silica surface with the linker APTES was performed. Finally the link of the zein protein to the particle’s surface via the formation of amide bonds with the APTES amine was established (Scheme 1). An additional zein-capped material loaded with the Rhodamine B dye (M41–RhB–Z) was also prepared.

**Figure 1:**
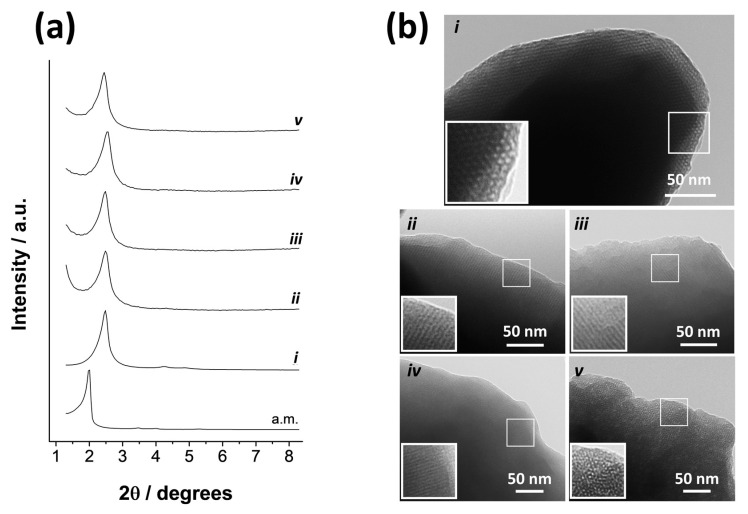
(**a**) Normalized X-ray patterns of all the synthesized solids. From bottom to top, the reported solids are as follows: “as made” material (a.m.), calcined support M41 (***i***) and final materials, M41–RhB–Z (*ii*), M41–Thy–Z (*iii*), M41–Car–Z (*iv*), M41–Cin–Z (*v*). (**b**) TEM images of M41 solids (*i*), M41–RhB–Z (*ii*), M41–Thy–Z (*iii*), M41–Car–Z (*iv*), M41–Cin–Z (*v*). Insets correspond to a 2× magnification of the framed areas.

The synthesized solids were characterized using standard techniques. Normalized X-ray patterns of all the solids (MCM-41 “as made”, calcined M41, M41–RhB–Z, M41–Thy–Z, M41–Car–Z and M41–Cin–Z) are shown in Figure 1a. The diffractogram of the MCM-41 “as made” shows four low-angle peaks characteristic of a hexagonal pore arrangement. The peaks were indexed from left to right as (1 0 0), (1 1 0), (2 0 0) and (2 1 0) Bragg reflections, respectively. After calcination, a notable shift towards higher 2θ values compared with the “as made” material can be observed. This phenomenon is related with cell contraction due to the condensation of silanol groups in the calcination process. It can also be appreciated that the reflections (1 1 0), (2 0 0) and (2 1 0) almost disappeared in the curves corresponding to the gated solids. This fact is due to the large amount of organic matter contained in the final solids, which causes partial loss of the structural order. However, the clear presence of the (1 0 0) peak in all the diffractograms demonstrates that neither the calcination nor the loading and functionalization processes modify the mesoporous structure.

The expected porous structure of the starting M41 solid was also confirmed through TEM micrographs (Figure 1b). In these images, the typical matrix of the MCM-41 material composed of narrow channels with hexagonal arrangement can be appreciated. This structure is visualized as alternating white and black stripes, and the pore stacking is visible when the pores are seen frontally in the image. Comparing the images of the final solids (Figure 1b(*ii*–*v*)) with the M41 one (Figure 1b(*i*)), it is possible to see structure preservation after the loading and functionalization processes even despite the loss of contrast in the images due to the presence of organic matter.

**Figure 2:**
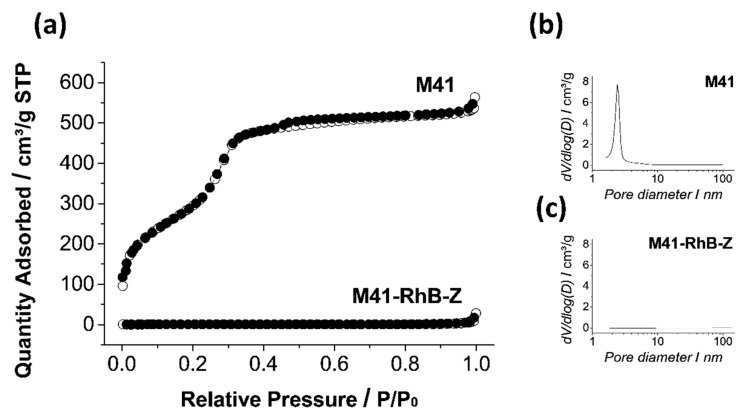
(**a**) Nitrogen adsorption (○)–desorption (●) isotherms of the calcined M41 solid and the final M41–RhB–Z solid at standard temperature and pressure conditions (STP); (**b**) pore distribution graph for M41 and (**c**) pore distribution graph for M41–RhB–Z.

The N_2_ adsorption-desorption isotherms of the calcined support (M41) are shown in Figure 2a. A typical curve for this mesoporous solid consisting of an adsorption step in the interval of P/P_0_ values between 0.1–0.3 can be observed. The curve fits with type IV isotherm, in which the increase produced in gas absorption corresponds to condensation of N_2_ molecules within the mesopores of the inorganic structure [50]. The absence of the hysteresis loop in this interval and the narrow BJH distribution of pore diameters (Figure 2b) suggest the existence of uniformly cylindric mesopores in which gas adsorption and desorption processes are carried out by the same mechanisms. The N_2_ adsorption-desorption isotherm of M41–RhB–Z solid is typical of mesoporous systems with practically filled mesopores (see Figure 2a). The high amount of organic matter in the solid prevents the adsorption of gas molecules, so the registered curve is completely flat when compared with the one of the empty starting material. Consequently, the absence of appreciable mesoporosity is observed (see Figure 2c), and a relatively low quantity of adsorbed N_2_ and low surface area (see Table 1) were obtained. Solids containing EOCs loaded into the pores could not be analyzed by this technique due to the previous degassing process under high vacuum and high temperature applied to the samples that would extract the encapsulated molecules.

**Figure 3:**
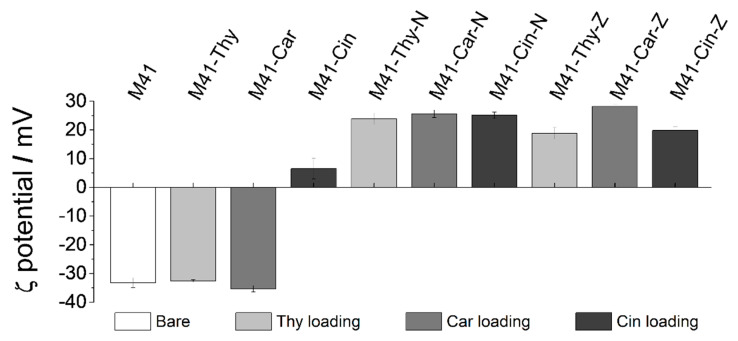
ζ potential values of the solids in the different synthesis steps.

The different stages of the synthesis process were followed by the ζ potential. The obtained values are shown in Figure 3. As it can be observed, a starting negative ζ value was obtained for the bare material (M41), which is related to the deprotonated silanol groups on the particle’s surface. The negative charge of M41 particles was maintained after loading with Thy and Car. A remarkable fact was the slightly positive value of the M41–Cin solid, possibly due to the formation of hemiacetal groups between the aldehyde of Cin and the silanolate groups on the MSP surface, which compensates for the negative charge on the M41 surface. This process did not occur with the other two EOCs (Thy and Car) since their active groups are alcohols, which do not react with silanolates. The hemiacetal formation is a pH-dependent reversible phenomenon [51], so the interaction of Cin with silanolates can be reverted when APTES is added. APTES-functionalization occurred effectively as is seen in the value of the ζ potential reached by M41–Cin–N which is comparable to the other M41–EOC–N values. Finally, only small changes in the ζ potential values were observed when the zein protein was attached to the external surface of the particles.

To determine the EOC payload content in M41–EOC–Z, DMSO cargo extraction was performed. This procedure allowed determining the payload within the different supports (μg EOC/mg M41–EOC–Z) and the obtained values are shown in Table 2. As can be seen, the encapsulation efficiency of the M41–Cin–Z solid was greater than that obtained with the other employed EOCs, i.e., Thy and Car.

**Figure 4:**
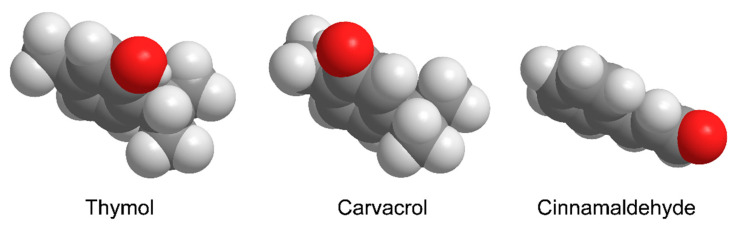
ChemDraw 3D MM2 energy-minimized model showing the 3D structure of thymol, carvacrol and cinnamaldehyde.

The differences in cargo loading efficiency may be due to the different structure of the studied molecules. The three-dimensional structure of the loaded EOCs is evidenced by the MM2 energy-minimized ChemDraw 3D model shown in Figure 4. As it can be seen in this Figure, Thy and Car molecules are isomers with notably higher steric hindrance than the Cin molecule, which is flat. This flat structure in aromatic compounds favors π–π interactions, which permits the accommodation of a greater number of molecules in a given space and might explain the larger loading of Cin in comparison with Thy and Car.

### 2.2. Controlled Cargo Release

**Figure 5:**
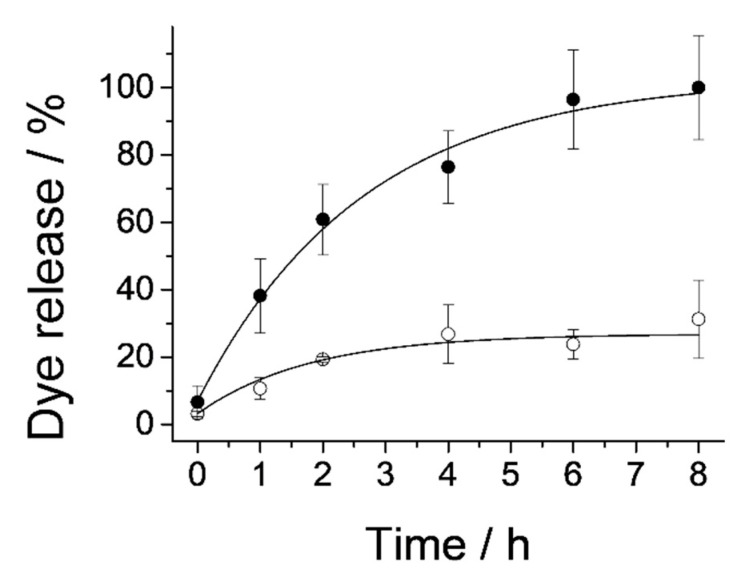
Cargo release profiles of RhB from M41–RhB–Z in H_2_O at pH 8 (○) and when proteolytic enzymes (pronase) are present (●). Release assays were performed in triplicate, and error bars correspond to the standard deviation.

Release assays were carried out with M41–RhB–Z to confirm the mechanism of the protein-gated support to modulate cargo release in the presence of proteolytic enzymes in the medium. Delivery studies from M41–RhB–Z were performed in H_2_O at pH 8 in the absence and in the presence of the pronase, a generic cocktail of proteolytic enzymes. As it can be seen in Figure 5, the cargo released from the system when no proteolytic enzymes were present was relatively low compared to the cargo delivered in the presence of the pronase. This confirms that cargo delivery of the chosen dye is hindered by the zein protein anchored to the surface of the mesoporous silica particles while the presence of proteolytic enzymes favors the sustained release of the entrapped molecule over time.

**Figure 6:**
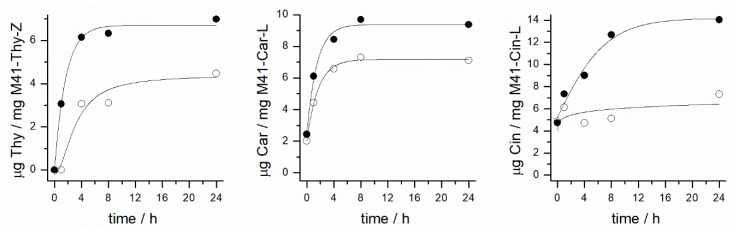
Cargo release profiles of EOCs from M41–Thy–Z (**left**); M41–Car–Z (**center**) and M41–Cin–Z (**right**) in H_2_O at pH 8. (○) in the absence and (●) in the presence of proteolytic enzymes (pronase).

To confirm the ability of the zein protein to also regulate the EOC payload delivery from M41–EOC–Z, release assays were carried out with the three synthesized materials, M41–Thy–Z, M41–Car–Z and M41–Cin–Z, following the procedure included in Section 3.7. As it can be seen in Figure 6, the highest release is obtained with the Cin solid and, although an initial release both in the absence and in the presence of the pronase was measured, the release of the M41–Cin–Z solid in the absence of the proteases was kept at a low basal level during the 24 h of the experiment.

### 2.3. Antimicrobial Activity of Free and Encapsulated EOCs

**Figure 7:**
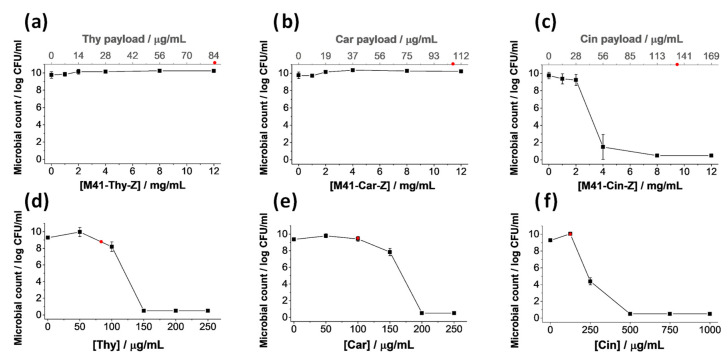
*E. coli* counts after incubation with M41–EOC–Z systems (M41-Thy-Z (**a**), M41–Car–Z (**b**) and M41–Cin–Z (**c**)) and free EOCs (thymol (**d**), carvacrol (**e**) and cinnamaldehyde (**f**)) according to particle and EOC concentration. Red dots mark the coincidental concentrations between the encapsulated EOC and the free molecule. Antimicrobial assays were performed in triplicate, and error bars correspond to the standard deviation.

The antimicrobial activity of free and encapsulated EOCs against *E. coli* as the model foodborne strain was evaluated by in vitro assays. The effectiveness of the studied systems evaluated by means of *E. coli* microbial counts after 24 h of treatment with the antimicrobials is shown in Figure 7. Different concentration ranges were tested for each EOC in accordance with previously reported studies [52].

All free EOCs were able to completely inhibit bacterial growth in the tested concentration ranges (50–250 μg/mL for Thy and Car and 125–1000 μg/mL for Cin), specifically, the MBC obtained for the free EOCs was found to be in the following concentration ranges: 100–150 μg/mL for Thy, 150–200 μg/mL for Car and 250–500 μg/mL for Cin. These results are in agreement with the data previously described in the literature, where the studied EOCs were reported to have MBC values against *E. coli* of 240–300.5 μg/mL for Thy, 244.2–366.4 μg/mL for Car and 262.5 μg/mL for Cin [53]. It can be observed that among the tested free EOCs, the least active was cinnamaldehyde, since a higher concentration was needed to obtain the same inhibitory effect (see Figure 7d for Thy, Figure 7e for Car and Figure 7f for Cin antimicrobial activities).

In the case of M41–EOC–Z microdevices, the maximum concentration of 12 mg/mL was chosen since a higher particle concentration would not be feasible in a real application. In Figure 7a–c, the bactericidal effect of the M41–EOC–Z solids is shown. In order to relate the bactericidal effects of the microdevices to those of the free EOCs, the coincidental concentration data in the Figure 7 top and bottom graphs were highlighted with red dots (coincidental data are based on the maximum EOC payload released obtained during the EOC release measurements, see Section 2.2).

In Figure 7a,b, it can be appreciated that the M41–Thy–Z and M41–Car–Z systems did not reach MBC at any of the tested concentrations. This circumstance might be explained by the fact that the payload released by these gated microdevices is below or very close to the MBC of the corresponding free compound. In contrast, the cinnamaldehyde-loaded solid showed a clear bactericidal effect, achieving the maximum effect at a particle concentration of 8 mg/mL, which corresponds to a Cin payload of 113 μg/mL. The Figure also shows that the M41–Cin–Z solid was able to significantly decrease the microbial population to a concentration as low as 4 mg/mL, which corresponds to a Cin payload of 50 μg/mL. These results indicate that the M41–Cin–Z system is not only better than the other studied M41–EOC–Z, but also has a greater bactericidal effect than free Cin. The MBC of the M41–Cin–Z system falls within the 56–113 μg of the Cin/mL range. This concentration is much lower than the MBC of free Cin, which, as stated before, was in the 250–500 μg/mL range. This MBC value obtained is also much lower than those reported in other previous works in which this compound was tested against different microorganisms [53,54,55,56]. This MBC improvement can be related to the advantages offered by encapsulation and active release of this volatile compound from gated MSP systems. In this way, the design proposed for this microdevice allows the local concentration of EOCs to be increased and prevents its evaporation from the medium, achieving the same antimicrobial effect with a lower active compound concentration.

**Figure 8:**
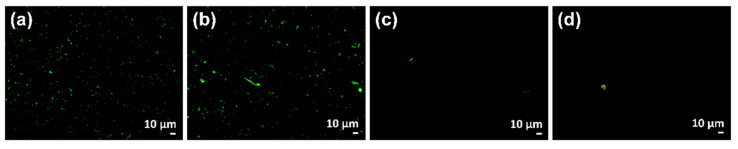
Fluorescence images of untreated *E. coli* (**a**) and the cells treated with M41–Z (**b**) as the negative control, free Cin (**c**) and M41–Cin–Z (**d**) after 5 h of incubation. The study was performed using a two-color fluorescent LIVE/DEAD^®^ *Bac*Light™ assay used to visualize viable (green) and dead (red) bacteria. Red-stained bacteria are not visible when dead cells do not remain intact after treatment.

Beside plate counting results, the bacterial viability of the cells treated with the free and encapsulated Cin was analyzed with a two-color fluorescent LIVE/DEAD^®^ *Bac*Light™ kit in order to visualize the remaining viable cells (see Section 3.8.2 for details). As an outline of the kit’s performance, living cells become green-stained and dead bacteria that remain physically intact in the medium are red-stained, thus allowing qualitatively visualizing cell viability. With this aim, cells incubated under different conditions (without treatment (positive control), with unloaded M41–Z particles (negative control), with free Cin and with M41–Cin–Z) were stained with the aforementioned kit (see Section 3.8.2 for details). Afterwards, fluorescence microscopy images were obtained. The resulting micrographs are shown in Figure 8. As it can be seen, there was no difference between positive (Figure 8a) and negative controls (Figure 8b) with regard to the *E. coli* viability (green cells). In contrast, the number of living cells (shown in green color) was practically reduced to zero when free Cin (Figure 8c) or M41–Cin–Z (Figure 8d) was added to the medium at the tested concentrations. The absence of red-stained cells is related to the absence of intact dead cells. This suggests cell envelope disruption and leakage of intracellular content resulting in the loss of cellular structure and the inability of the red fluorophore to stain these cells. These results are in accordance with the previous studies using essential oil components as antimicrobials [57,58].

## 3. Materials and Methods

### 3.1. Reagents, Bacterial Strain and Culture Media

Tetraethyl orthosilicate (TEOS), triethanolamine (TEAH_3_), sodium hydroxide (NaOH), *N*-cetyltrimethylammonium bromide (CTAB), (3-aminopropyl)triethoxysilane (APTES), *N*-(3-dimethylaminopropyl)-*N*′-ethylcarbodiimide (EDC), trifluoroacetic acid (TFA), rhodamine B (RhB), thymol (Thy), carvacrol (Car) and cinnamaldehyde (Cin) were provided by Sigma-Aldrich (Sigma-Aldrich Química S.L., Madrid, Spain). Alpha-zein (corn protein) (zein), ethanol (extra pure), methanol (MeOH), acetonitrile (ACN), hexane and dimethyl sulfoxide (DMSO) were purchased from Scharlab (Barcelona, Spain). *Escherichia coli* K12 (CECT 433) was obtained from the Spanish Type Culture Collection (CECT, Burjassot, Spain). The bacterial strain was reconstituted according to the CECT instructions. After that, the bacterial stock was maintained at 4 °C in a plate count agar before its use. *E. coli* inoculum was prepared by placing one single colony of the *E. coli* strain into 10 mL of tryptic soy broth (TSB). The mixture was incubated at 37 °C for 24 h in order to obtain an inoculum with a density of approximately 10^8^ cells/mL of broth. All culture media were supplied by Scharlab S.A. (Barcelona, Spain).

### 3.2. Inorganic Support Synthesis

The synthesis of the mesoporous material was performed according to the protocol described by Bernardos et al. [59]. The synthesis process was carried out following the “atrane route” [60] in which the structure-directing agent is composed of an intermediate species between CTAB and TEAH_3_. The molar ratio between the reagents was as follows: 7 TEAH_3_: 2 TEOS: 0.52 CTAB: 0.5 NaOH: 180 H_2_O.

For the microparticle synthesis, 0.49 g of NaOH were added to 25.79 g of TEAH_3_ and the mixture was heated to 120 °C in order to evaporate the ethanol generated in the condensation reaction. Then, the mixture was cooled to 70 °C. TEOS in the amount of 11 mL was added dropwise and the mixture was heated again to 120 °C while stirring. Once the temperature was reached, 4.68 g of CTAB were added, and the mixture was cooled again to 70 °C. Finally, 80 mL of distilled H_2_O were added, and the mother liquor was stirred vigorously for 1 h at room temperature. The final mixture was transferred to a Teflon flask and kept at 100 °C for 24 h. After that, the suspension was filtered under vacuum and washed until neutral pH. The material was dried and finally calcined at 550 °C to completely remove the surfactant molecules that conformed the nanotubular template. Following this procedure, the final MCM-41 microparticles, bare and unloaded (M41 solid), were obtained.

### 3.3. Synthesis of Gated Microdevices

#### 3.3.1. Synthesis of an M41–RhB-Gated System

RhB was encapsulated into M41 microparticles by the immersion method following a similar loading procedure to the one carried out in previous works [61]. With that purpose, 1 g of M41 was added to a solution of RhB (0.8 mmol in 40 mL of H_2_O) and stirred for 24 h. The mixture was filtered and the resulting solid, M41–RhB, was dried under vacuum. Once M41 was loaded with RhB, the surface of the support was functionalized with the zein protein. For the protein bonding, previous surface functionalization with APTES as a linker between the protein and the MSP–silanol groups was necessary. Functionalization was performed by adding 8 mL APTES to a suspension of 1.2 g of the loaded solid in 25 mL of H_2_O. The mixture was stirred for 5.5 h at room temperature. The resulting APTES-functionalized particles (M41–RhB–N) were collected by centrifugation and dried under vacuum. Finally, the binding of zein (corn protein) on the surface of M41–RhB–N was performed through a covalent amide bond between carboxylate moieties in the protein and amino groups from APTES following similar protocols to those described in the literature with slight modifications [34]. In a typical procedure, a suspension of 1 g of zein in 30 mL of H_2_O and 200 mg of EDC was prepared and stirred for 30 min. Then, 700 mg of M41–RhB–N were added and the mixture was stirred at room temperature overnight. The final solid was collected by centrifugation and washed three times with mixtures of ethanol/water gradually increasing the proportion of water. Finally, the protein-functionalized solid (M41–RhB–Z) was dried under vacuum.

#### 3.3.2. Synthesis of M41–EOC-Gated Systems

Three different EOCs, thymol, carvacrol and cinnamaldehyde (Thy, Car and Cin, respectively), were loaded into the mesoporous material by steam adsorption by mixing 250 mg of M41 solids and 250 mg of each EOC in a perfectly closed vial. The mixtures were shaken at 40 °C for 24 h until the added amount of EOC was completely adsorbed and the solids (i.e., M41–EOC: M41–Thy, M41–Car and M41–Cin, respectively) looked completely dry. Once M41 was loaded with the different cargo molecules, the surface of the support was functionalized with the zein protein following a similar procedure as described above. In a typical procedure, 8 mL APTES was added to a suspension of 1.2 g of the loaded solid in 25 mL of H_2_O. The mixture was stirred for 5.5 h at room temperature. The resulting APTES-functionalized particles (M41–EOC–N) were collected by centrifugation and dried under vacuum. A suspension of 1 g of zein in 30 mL of H_2_O and 200 mg of EDC was prepared and stirred for 30 min. Then, 700 mg of M41–EOC–N were added to the mixture and stirred at room temperature overnight. The final solids were collected by centrifugation and washed three times with mixtures of ethanol/water gradually increasing the proportion of water. Finally, the protein-functionalized solids (M41–EOC–Z: M41–Thy–Z, M41–Car–Z and M41–Cin–Z) were dried under vacuum.

#### 3.3.3. Synthesis of an Unloaded Gated System

Additionally, a solid without loaded molecules but gated with the zein protein (M41–Z) was synthesized and used as the negative control in the microbiological tests. For this purpose, 500 mg of M41 were functionalized with APTES (3 mL) as described above obtaining the M41–N solid. The final unloaded gated system, M41–Z, was obtained by binding the zein protein (150 mg) to the amino-terminal groups in M41–N (50 mg) following the same procedure as previously described.

### 3.4. Characterization Methods

Powder X-ray diffraction (PXRD), transmission electron microscopy (TEM), N_2_ adsorption–desorption isotherms, ζ potential, thermogravimetric analysis (TGA) and fluorescence spectroscopy were used to characterize the synthesized solids. PXRD was performed on a Bruker D8 Advance diffractometer (Bruker, Coventry, UK) using Cu Kα radiation. TEM images were obtained with a JEOL JEM-1010 (JEOL Europe SAS, Croissy-sur-Seine, France). N_2_ adsorption–desorption isotherms were recorded with a Micromeritics TriStar II Plus automated analyzer (Micromeritics Instrument Corporation, Norcross, GA, USA). Inorganic samples were degassed at 120 °C in vacuum overnight. Samples with organic content were degassed at 70 °C overnight. The specific surface areas were calculated from the adsorption data in the low pressure range using the BET (Brunauer Emmett Teller) model. Pore size was determined following the BJH (Barclay James Harvest) method. To determine the ζ potential of all the solids, Zetasizer Nano ZS equipment (Malvern Instruments, Malvern, UK) was used. Samples were dispersed in distilled water at a concentration of 1 mg/mL. Before measuring, each sample was sonicated for 5 min to preclude aggregation. The particle mobility values were used to calculate ζ values by applying the Smoluchowski model. Each sample was measured in triplicate at 25 °C, performing three recordings per measurement. The average of the nine obtained values was reported as the ζ potential, and error bars represent the standard deviation value. Fluorescence spectroscopy measurements were performed with a JASCO FP-8300 spectrofluorometer (JASCO, Easton, PA, USA).

### 3.5. EOC Payload Quantification

To determine the payload content in M41–EOC–Z microdevices, a DMSO extraction process was performed. For this purpose, 2 mg of the corresponding solid were suspended in 2 mL of DMSO and the mixture was kept under stirring for 24 h. After that, the EOC payload released was quantified by measuring the absorbance of the sample at 280 nm.

### 3.6. RhB Release Assay

For the cargo release experiment, 5 mg of the final solid (M41–RhB–Z) were placed in 10 mL of H_2_O at pH 8, and the other 5 mg of M41–RhB–Z were placed in 10 mL of a pronase enzyme solution (0.12 mg/mL of pronase in H_2_O at pH 8). At certain times, aliquots were taken and filtered. The RhB delivery from the pore voids to both solutions was quantified via the fluorescence emission band of this molecule at 572 nm (excitation at 555 nm). The release assay was performed in triplicate.

### 3.7. EOC Release Assay

For the EOCs’ cargo release experiment, 1 mg of each of the final solids (M41–EOC–Z) was placed in 1 mL of H_2_O at pH 8, and the other 1 mg of M41–EOC–Z was placed in 1 mL of a pronase enzyme solution (0.12 mg/mL of pronase in H_2_O at pH 8). At certain times, aliquots were taken and the amount of EOC released was quantified by high-performance liquid chromatography (HPLC) in the case of carvacrol and by gas chromatography–mass spectrometry (GC–MS) in the case of thymol and cinnamaldehyde.

To perform Car release experiments, the collected aliquots were centrifuged to remove the suspended microparticles. The proteins present in the supernatants were precipitated with MeOH (1 mL) at −14 °C, and the mixture was then centrifuged. Car delivery from M41–Car–Z to the supernatant solution was analyzed by HPLC with a Novapack C18 column (Waters Alliance^®^ e2695, Milford, MA, USA). The mobile phase used was ACN:H_2_O (50:50 *v*/*v*) with 0.05% of TFA and the absorbance detector used the fluorescence wavelength (λex) of 280 nm. Two hundred mL were injected and a flow of 1 mL/min was fixed. The methods were previously validated showing adequate linearity, precision and accuracy (R > 0.99 and coefficient of variation <5%).

In the case of the Thy and Cin cargo release experiment, each of the collected aliquots was shaken with 1 mL of hexane to extract the corresponding EOC and perform its quantification by GC–MS. Then, 2 μL of each extracted sample were directly introduced into the injector. The analysis was performed in a 6890/5975 inert GC–MS (Agilent Technologies, Santa Clara, CA, USA) equipped with an HP-5 fused silica capillary column (30 m × 0.25 mm × 0.25 μm). Helium gas (ultrahigh purity grade, 99.999%) was used as the carrier gas at a constant flow rate of 1 mL/min. The oven temperature was programmed initially at 60 °C for 3 min with a following ramp from 60 to 100 °C at a heating rate of 10 °C/min; the oven temperature was then further increased to 140 °C at 5 °C/min and finally increased again to 240 °C at a rate of 20 °C/min; the hold time was 27 min. The injector and MS transfer line temperatures were set at 250 °C and 230 °C, respectively. The MS analysis parameters were the electron ionization (EI) source, electron energy of 70 eV, solvent delay of 3 min and m/z of 40–550 amu. Thy and Cin were identified according to their retention index and by matching mass spectra with the standard mass spectra from the National Institute of Standards and Technology (NIST) MS Search 2.0 library. In order to develop calibration methodology for our study, Thy and Cin main peak areas from GC–MS were plotted as a function of the concentration of Thy and Cin in hexane (from 5 to 250 μg/mL). A linear trend was observed.

### 3.8. Microbiological Analysis

#### 3.8.1. Antimicrobial Susceptibility Assays

The minimum bactericidal concentration (MBC) of the antimicrobial compounds was obtained using the macrodilution method [62]. The MBC was defined as the lowest concentration of the antimicrobial compound that would kill 99.9% of *E. coli* cells. The antimicrobial assay was performed in a liquid medium to allow the contact between bacteria and the free or encapsulated antimicrobials during incubation, followed by plating to enumerate the remaining viable colony-forming units (CFUs). Different compound concentrations were prepared in Erlenmeyer flasks with 15 mL of TSB for each free EOC in accordance with previous studies considering the obtained MBC values in those works [52,53]. The tested concentrations were 50, 100, 150, 200 and 250 μg/mL for Thy and Car and 125, 250, 500, 750 and 1000 μg/mL for Cin. Then, Erlenmeyer flasks, including the controls containing only the TSB medium, were inoculated with 10 µL of the *E. coli* suspension to obtain a final concentration of 10^6^ cells/mL. Finally, all the flasks were incubated with orbital stirring (150 rpm) at 37 °C for 24 h. In order to enumerate the viable cells after the antimicrobial treatment, decimal serial dilutions were prepared in sterile distilled water and 100-µL aliquots thereof were spread on Tryptone Bile X-glucuronide (TBX) plates. After plate incubation at 37 °C for 24 h, the colonies were counted, and the obtained results were expressed as log CFU/mL. To perform the M41–EOC–Z antimicrobial assays, the synthesized particles were added directly to the TSB contained in the Erlenmeyer flasks to obtain suspensions of each M41–EOC–Z microdevice at concentrations of 0, 1, 2, 4, 8 and 12 mg/mL, and the subsequent procedure was the same as that followed for free antimicrobials (vide supra). Positive controls, where the *E. coli* inoculum was allowed to grow without alterations, and negative controls, where the non-treating system M41–Z was added at the maximum particle concentration (12 mg/mL), were also performed. All the treatments (which include controls, free EOCs and M41–EOC–Z microdevices) were tested in triplicate.

#### 3.8.2. Determination of Bacterial Viability and Agglomeration by Fluorescence Assay

To develop this experiment, suspensions of bacteria-inoculated TSB with a final concentration of 10^6^ cells/mL were prepared and incubated under different conditions for 5 h. The studied conditions included bacteria treated with free Cin (400 μg/mL) or M41–Cin–Z (4 mg/mL) and also positive and negative controls (non-treated bacteria or bacteria treated with 4 mg/mL M41–Z, respectively). Treatment concentrations were chosen based on the microbial count results (see Section 3.8.1) with the aim of testing concentrations where both live and dead cells could be visualized in the same sample. The cells in each suspension were stained with a two-color fluorescent kit, LIVE/DEAD^®^ BacLight™ (Invitrogen, ThermoFisher Scientific, Renfrew, UK), used for visualizing the viable and remaining dead bacteria according to membrane cell integrity. The two-color kit is composed of SYTO 9 (green fluorescent nucleic acid stain) that labels all the microbial cells with either intact or damaged membranes and propidium iodide (red fluorescent nucleic acid stain) that passes only across damaged membranes, so it labels only the dead cells that remain present in the media. Propidium iodide reduces SYTO 9 fluorescence when both dyes coexist in the same cell, resulting in red labelling. This results in a green/red labeling of the living/dead cells, respectively. For that, 0.8 μL of the aforementioned kit (SYTO 9 and propidium iodide mixed at a ratio 1:1) were added to 500 μL of each studied suspension. The mixture was incubated in the dark for 10 min to allow the penetration of dyes. Then, 5 μL of stained cells were placed over poly-L-lysine-covered slides (Sigma-Aldrich, Madrid, Spain) and sealed with a coverslip. The samples were finally observed under a Motic BA310E trinocular microscope equipped with an Epi-Led module with a 3W Led 470 nm illuminator bulb, MB barrier filter (AT480/30×, AT505DC and AT515LP fluorescence filters) and a Moticam 3+ camera. The obtained fluorescence images were acquired on a dark field with a 100× objective and have a pixel size of 0.156 µm (64 px = 10 µm).

## 4. Conclusions

In this work, a new antimicrobial device applying for the first time the zein protein as a gating moiety and based on MSPs loaded with essential oil components is developed for the controlled release of natural antimicrobial compounds (essential oil components (EOCs) thymol, carvacrol and cinnamaldehyde). The antimicrobial action of cinnamaldehyde encapsulated inside the microdevice against *E. coli* is demonstrated by culturability and viability assays. Indeed, the results show not only a good inhibitory effect of cinnamaldehyde when encapsulated in gated MSPs, but also the enhancement of the cinnamaldehyde bactericidal effect when compared with the corresponding free compound. The accomplishment of this microdevice is based on the proteolytic secretion by bacteria acting as a triggering stimulus that allows the sustained release of the entrapped EOCs. In this way, the developed microdevice improves the antimicrobial properties of an essential oil component by decreasing its volatility and, hence, by increasing its local concentration. The developed antimicrobial system based on the combination of food grade molecules and a biocompatible support might be used as a new preservative in the food industry.

## Figures and Tables

**Table 1:** BET specific surface values, pore volumes and pore sizes calculated from the N_2_ adsorption–desorption isotherms for selected materials.

	SBET (m^2^ g^−1^)	Pore Volume ^a^ (cm^3^ g^−1^)	Pore Size ^a,b^ (nm)
M41	1108	0.91	2.50
M41–RhB–Z	1.71	0.01	-

^a^ Pore volumes and pore sizes are restricted to intraparticle mesopores. ^b^ Pore size was estimated by using the BJH model applied to the adsorption branch of the isotherm.

**Table 2:** Extracted content of cargo in the final solids (μg EOC/mg M41–EOC–Z).

	M41–Thy–Z	M41–Car–Z	M41–Cin–Z
μg EOC/mg M41–EOC–Z	11.5	6.2	18.1

## Data Availability

Not applicable.

## Abbreviations

MSPs	Mesoporous silica particles
EOCs	Essential oil components
Thy	Thymol
Car	Carvacrol
Cin	Cinnamaldehyde
Zein	α-zein corn-protein
PXRD	Powder X-ray diffraction
TEM	Transmission electron microscopy
TGA	Thermogravimetric analysis
